# Schistosomiasis in Saudi Arabia (2002–2024): A National Analysis of Trends, Regional Heterogeneity, and Progress Toward Elimination

**DOI:** 10.3390/tropicalmed11010025

**Published:** 2026-01-16

**Authors:** Yasir Alruwaili

**Affiliations:** 1 Department of Clinical Laboratory Sciences, College of Applied Medical Sciences, Jouf University, Sakaka 72388, Saudi Arabia; ysalruwaili@ju.edu.sa; 2 Center for Health Research and Innovations, Deanship of Graduate Studies and Scientific Research, Jouf University, Sakaka 72388, Saudi Arabia

**Keywords:** schistosomiasis, neglected tropical diseases, elimination, joinpoint regression, ARIMA, Saudi Arabia, surveillance

## Abstract

Schistosomiasis remains a major neglected tropical disease globally and presents particular challenges for countries transitioning from control to elimination. Saudi Arabia represents a unique epidemiological setting, having shifted from historical endemic transmission to very low reported incidence, yet long-term national analyses remain limited. A retrospective longitudinal analysis of national schistosomiasis surveillance data from 2002 to 2024 was conducted to evaluate temporal trends, clinical subtypes, regional distribution, and demographic characteristics. Joinpoint regression was used to identify significant changes in temporal trends, and autoregressive integrated moving average (ARIMA) models were applied to forecast national and regional trajectories. National incidence declined markedly from 5.5 per 100,000 in 2002 to 0.12 per 100,000 in 2024, with a notable change around 2010, followed by sustained low-level incidence. Intestinal schistosomiasis accounted for most cases, with increasing concentration among adult non-Saudi males and near-elimination among children. Regionally, cases were confined to a limited number of western and southwestern regions, particularly Ta’if, Al Baha, Jazan, and Madinah. Forecasting analyses indicated continued low-level detection without evidence of national resurgence. These findings demonstrate a transition to an elimination-maintenance phase and highlight the need for sustained surveillance in historically endemic regions and mobile populations.

## 1. Introduction

Schistosomiasis is a chronic parasitic disease of major public health importance and remains one of the World Health Organization’s priority neglected tropical diseases [1]. It is caused by trematode worms of the genus *Schistosoma* and is transmitted through contact with freshwater contaminated by cercariae that are released from infected intermediate host snails [2]. Human infection occurs during routine water-contact activities, while transmission is maintained through specific snail–parasite–environment interactions that define local ecological suitability [1,3]. Although sustained control efforts have resulted in significant global reductions in prevalence and morbidity, schistosomiasis continues to pose challenges in countries transitioning from control to elimination, where transmission becomes focal, case numbers are low, and surveillance data require careful interpretation [3,4]. In such settings, distinguishing sustained progress from residual transmission or re-emergence is essential for guiding elimination-maintenance strategies.

Globally, schistosomiasis remains a significant public health concern, with an estimated 200 million people currently infected and more than 700 million living in areas at risk of transmission [1]. As an increasing number of countries enter low-burden or post-control phases, strategic priorities have shifted from morbidity reduction toward interruption of transmission and sustained elimination [3]. In this context, long-term surveillance data, impact assessment, and robust analytical methods are increasingly important for evaluating progress and informing targeted interventions [4].

Saudi Arabia represents a distinctive epidemiological setting for schistosomiasis. Historically, both intestinal and urogenital schistosomiasis, caused primarily by *Schistosoma mansoni* and *Schistosoma haematobium*, respectively, were endemic in the Kingdom. They concentrated particularly in western and southwestern regions where freshwater systems supported suitable intermediate host snails [5,6,7,8,9]. Transmission of *S. mansoni* is associated with *Biomphalaria* snail species, mainly *Biomphalaria pfeifferi*, whereas *S. haematobium* transmission involves *Bulinus* species, including *Bulinus truncatus*, *B. beccarii*, and *B. wrighti*, which thrive in irrigation channels, slow-moving streams, dams, and other freshwater habitats [7,9]. These ecological conditions historically facilitated sustained transmission in localized agricultural and rural settings. The two clinical forms differ in patterns of morbidity, exposure risk, and detectability, with important implications for surveillance sensitivity and interpretation of temporal trends as countries approach elimination thresholds [2,9].

In response to this burden, national reviews documented widespread transmission during the mid-20th century, prompting the establishment of structured schistosomiasis control programs beginning in the 1970s [8]. These programs incorporated preventive chemotherapy with praziquantel, snail control and environmental modification, improvements in water supply and sanitation, and health education, later integrated into primary health care services, resulting in a marked and sustained decline in disease burden [7,8].

Within the Gulf Cooperation Council region, Saudi Arabia’s experience is broadly consistent with that of neighboring countries that have achieved substantial reductions in schistosomiasis transmission. Oman provides a notable regional example, having documented a sustained decline in *S. mansoni* transmission and achieved elimination benchmarks through long-term national monitoring and surveillance [10]. Recent official reports indicating the absence of detected intestinal schistosomiasis cases over eight consecutive years highlight both the feasibility of elimination in the region and the importance of continued observance in historically endemic or ecologically suitable areas [11].

Although several studies have examined schistosomiasis in Saudi Arabia, important gaps remain in the characterization of long-term national trends. Previous investigations have largely focused on localized prevalence estimates, specific clinical presentations, or short-term retrospective analyses [12,13,14]. More recent studies and reviews have documented progress toward elimination; however, they have not consistently applied analytical approaches capable of identifying statistically significant change points in temporal trends or projecting future trajectories to support elimination-maintenance planning [15,16,17,18]. Therefore, the present study analyzes national schistosomiasis surveillance data in Saudi Arabia from 2002 to 2024 to provide a comprehensive assessment of long-term epidemiological trends. By integrating descriptive analyses with joinpoint regression to identify significant changes in temporal patterns and time-series forecasting to assess future trajectories, this study aims to support monitoring and evaluation, strengthen impact assessment, and inform targeted surveillance strategies as Saudi Arabia advances toward sustained schistosomiasis elimination.

## 2. Materials and Methods

### 2.1. Study Design and Data Source

The study employed a retrospective longitudinal design based on publicly available national schistosomiasis surveillance data in Saudi Arabia covering the period from 2002 to 2024. Data were obtained from officially reported annual records of the Saudi Ministry of Health (MoH), which compiles nationwide notifications of schistosomiasis cases as part of routine communicable disease surveillance activities [19].

The dataset included annual counts of confirmed schistosomiasis cases reported across the Kingdom, along with population-based incidence rates where available. Surveillance data were aggregated at the national and regional levels and did not contain individual-level identifiers, clinical records, or personal information.

### 2.2. Study Area and Regional Classification

**Figure 1:**
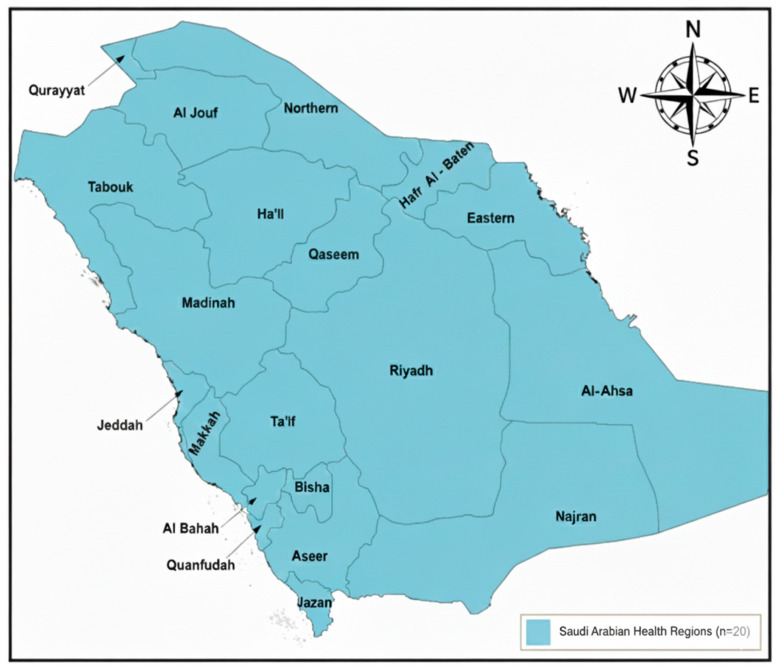
Ministry of Health (MoH) health regions of Saudi Arabia. The map illustrates the 20 MoH health regions (cities) used for public health surveillance and disease reporting in Saudi Arabia.

Saudi Arabia is administratively divided into 13 regions; however, public health surveillance and disease reporting by the Ministry of Health (MoH) are organized according to 20 health regions corresponding to major cities. Accordingly, all regional analyses in this study were conducted using the MoH health-region framework applied in routine schistosomiasis surveillance to ensure full alignment with official reporting practices. The geographic framework used for health-region classification is illustrated in Figure 1 and was applied consistently across all regional epidemiological analyses. The map presents the officially recognized 20 health regions and was prepared using publicly available boundary data, with health-region delineations and labels defined to reflect the MoH surveillance structure [19].

### 2.3. Case Definition and Classification

Reported schistosomiasis cases were classified according to the clinical form recorded in national surveillance reports. These included intestinal schistosomiasis, predominantly caused by *S. mansoni*; urogenital (urinary) schistosomiasis, caused by *S. haematobium*; and mixed infections, defined as cases with evidence of both intestinal and urinary involvement. Demographic variables extracted from surveillance records included sex, nationality (Saudi vs. non-Saudi), and age group (<5, 5–14, 15–39, and ≥40 years), where available. Subnational demographic and regional analyses were restricted to the period 2010–2024, reflecting improvements in the completeness and consistency of health-region reporting during this timeframe. Cases classified as non-Saudi include both non-citizen residents living in Saudi Arabia and visitors, as nationality status is recorded without distinction between residency and travel status. Individual-level information on duration of stay, travel history, or place of infection acquisition was not available in the aggregated dataset.

### 2.4. Descriptive Analysis

Descriptive analyses were performed to summarize national temporal trends, clinical subtype distribution, and demographic characteristics of reported schistosomiasis cases. Regional distributions were assessed using aggregated data from health regions with available reporting. Graphical and tabular visualizations were used to explore temporal, spatial, and demographic patterns in disease burden.

### 2.5. Temporal Trend Analysis

Long-term temporal trends in national schistosomiasis case counts were evaluated using joinpoint regression implemented through segmented linear models. Annual case counts were modeled as a function of calendar year, allowing the identification of a single joinpoint representing a statistically significant change in trend slope. The joinpoint location was estimated iteratively by minimizing model residuals, and fitted values were used to characterize temporal patterns before and after the identified breakpoint. This approach was selected to objectively assess trend transitions in elimination-phase surveillance data without imposing predefined breakpoints.

### 2.6. Time-Series Forecasting

Forecasting of national and regional schistosomiasis case counts was conducted using autoregressive integrated moving average (ARIMA) models applied to log-transformed annual case counts to stabilize variance [20]. Model selection was performed using an automated approach based on information criteria, as implemented in the *auto.arima()* function, which evaluates multiple candidate models and selects the most parsimonious specification that best fits the observed data [20]. Formal external or out-of-sample validation was not performed since the analysis was based on complete national surveillance time series rather than independent datasets. Instead, model adequacy was assessed internally through consistency between observed and fitted values and by explicitly quantifying forecast uncertainty using prediction intervals. Forecast uncertainty was quantified using both 80% and 95% prediction intervals to reflect plausible ranges of future case counts under continued surveillance conditions. All statistical analyses were conducted using R software (version 4.5.2) [21]. The *forecast* package was used for ARIMA model fitting and time-series forecasting [20], *segmented* for change-point (joinpoint) regression analysis [22], *ggplot2* for data visualization [23], and *sf* for handling and visualizing spatial data [24].

### 2.7. Statistical Software

Data management, preliminary cleaning, and validation were conducted using Microsoft Excel (Microsoft Corporation, Redmond, WA, USA). Statistical analyses, data visualization, segmented regression, and time-series modeling were performed using R statistical software (R Foundation for Statistical Computing, Vienna, Austria) within the RStudio integrated development environment (Posit Software, version 2026.01.0+392, Boston, MA, USA), employing standard and widely used R packages for descriptive analysis, joinpoint regression, and ARIMA modeling.

### 2.8. Data Availability

All data used in this study were obtained from publicly available annual surveillance reports published by the Saudi Ministry of Health. Aggregated national and regional datasets analyzed during the current study are available from the corresponding author upon reasonable request. No accession numbers apply, as the data are derived from routinely published governmental surveillance summaries [19].

### 2.9. Ethical Considerations

This study utilized aggregated, anonymized secondary surveillance data obtained from publicly available Ministry of Health records. No individual-level identifiers were accessed. Ethical approval was not required in accordance with national regulations governing the use of non-identifiable public health surveillance data.

## 3. Results

### 3.1. National Burden and Incidence of Schistosomiasis (2002–2024)

National schistosomiasis cases, incidence rates, clinical subtypes, and demographic characteristics from 2002 to 2024 are summarized in Table 1. A marked and sustained decline in national schistosomiasis burden was observed over the study period. In 2002, a total of 1159 cases were reported nationwide, corresponding to an incidence of 5.5 cases per 100,000 population. Annual case counts declined steadily over subsequent years, reaching 120 cases (0.50 per 100,000 population) by 2010.

From 2011 onward, national incidence remained consistently below 1 case per 100,000 population, despite minor year-to-year fluctuations. During this period, annual reported cases ranged from a minimum of 19 cases in 2022 (0.06 per 100,000 population) to a maximum of 320 cases in 2013 (1.07 per 100,000 population). In the most recent surveillance year (2024), 43 cases were reported nationally, corresponding to an incidence of 0.12 per 100,000 population. Overall, the national time series demonstrates a transition from moderate endemic transmission in the early 2000s to sustained low-level case detection in recent years.

### 3.2. Temporal Trend and Joinpoint Analysis

**Figure 2:**
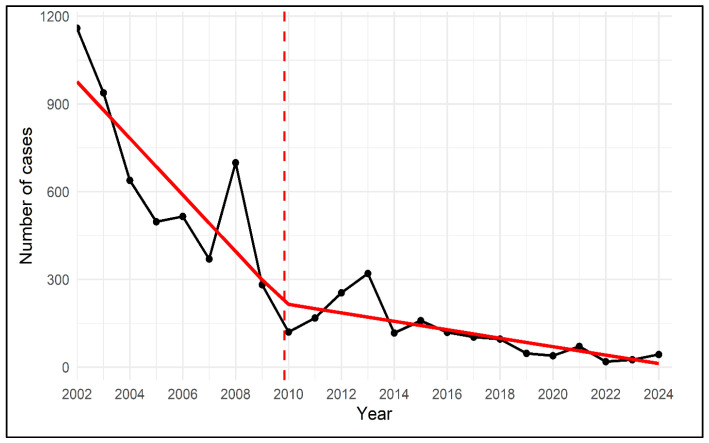
National trend of reported schistosomiasis cases in Saudi Arabia, 2002–2024, with joinpoint regression analysis. The solid black line represents observed annual case counts, while the solid red line indicates fitted values from segmented (joinpoint) regression. The dashed vertical red line denotes the estimated joinpoint year at which a statistically significant change in the slope of the temporal trend was identified, reflecting a transition in the rate of decline of reported cases.

National temporal trends in reported schistosomiasis cases are shown in Figure 2. Joinpoint regression analysis showed a statistically significant change in the national trend around 2010. Prior to this point, annual case counts declined gradually from 1159 cases in 2002 to 120 cases in 2010. Following the identified joinpoint, the decline became more pronounced and was followed by stabilization at low levels, with no sustained increase observed through 2024. No additional statistically significant joinpoints were detected across the study period.

### 3.3. Clinical Subtypes of Schistosomiasis

As shown in Table 1, intestinal schistosomiasis was the predominant clinical subtype throughout the study period. Between 2002 and 2024, intestinal schistosomiasis accounted for 4580 of 6301 reported cases (72.7%). Urogenital (urinary) schistosomiasis accounted for 2299 cases (36.5%), while mixed infections were uncommon, representing 32 cases (0.5%). Following 2010, intestinal schistosomiasis consistently accounted for the majority of reported cases, frequently exceeding 80% of annual notifications.

### 3.4. Demographic Characteristics of Reported Cases

Across the study period, a consistent male predominance was observed. Males accounted for 5051 of 6301 reported cases (80.2%), while females accounted for 1250 cases (19.8%). With respect to nationality, Saudi nationals accounted for 3136 cases (49.8%), while non-Saudi nationals accounted for 3165 cases (50.2%). The proportion of cases among non-Saudi nationals increased over time, rising from 42.2% (489/1159) in 2002 to 81.4% (35/43) in 2024.

Age-specific data available from 2006 onward indicated that schistosomiasis predominantly affected adults. Individuals aged 15–39 years accounted for 2912 cases, while those aged ≥40 years accounted for 634 cases. Pediatric cases were infrequent in later years, with 11 cases reported among children aged <5 years and 497 cases among those aged 5–14 years.

### 3.5. Temporal Patterns in National Indicators

**Figure 3:**
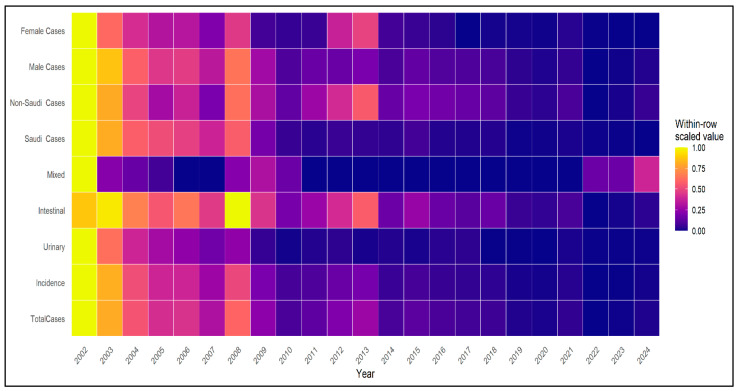
Heatmap of temporal patterns in schistosomiasis burden and demographic characteristics in Saudi Arabia, 2002–2024. Color intensity represents within-row scaled values, allowing visualization of relative temporal changes within each indicator.

Temporal patterns in schistosomiasis burden and demographic indicators are illustrated in Figure 3. Heatmap analysis showed higher case counts and incidence rates during the early years of surveillance, followed by substantially lower intensity after 2010. Demographic indicators demonstrated a shift toward adult age groups and non-Saudi populations over time, with minimal contribution from pediatric cases in later years.

### 3.6. Regional Distribution of Schistosomiasis (2010–2024)

**Figure 4:**
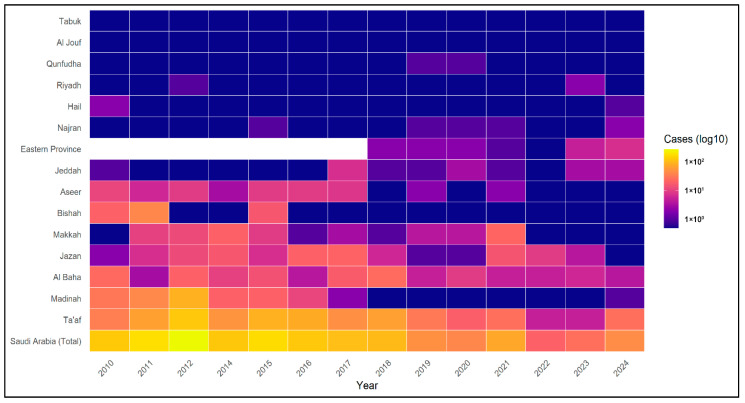
Regional heatmap of reported schistosomiasis cases in Saudi Arabia, 2010–2024. The heatmap displays annual reported case counts by region, with color intensity representing the logarithm (base 10) of case numbers to accommodate wide variation in magnitude across regions and years. Data for 2013 are not included due to incomplete regional reporting. Eastern Province data are shown from 2018 onward, reflecting the initiation of consistent regional surveillance reporting. National total case counts are included for reference.

Regional distributions of schistosomiasis cases are summarized in Table 2 and visualized in Figure 4. Marked geographical heterogeneity was observed across health regions from 2010 to 2024. The highest cumulative case counts were reported in Ta’if, Madinah, Al Baha, and Jazan, which together accounted for 1110 of 1924 regionally reported cases (57.7%). In contrast, several regions reported very low case counts over the same period. Riyadh and Hail, for example, reported only three cases each. Regions with no reported cases during the available reporting period were not included in the table or figure, such as Al-Qurayyat and the Northern Borders; however, Al Jouf and Tabuk were retained in the figure for comparative purposes.

### 3.7. Regional Comparison of Clinical and Demographic Patterns

Across higher-burden regions, intestinal schistosomiasis predominated. In Ta’if, intestinal cases accounted for 421 of 639 cases (65.9%), while in Madinah they accounted for 196 of 204 cases (96.1%). Male predominance was consistent across regions, exceeding 80% in most settings. Non-Saudi nationals constituted a considerable proportion of cases in urban regions such as Makkah (71.4%) and Jeddah (70.0%), whereas Saudi nationals accounted for the majority of cases in regions such as Jazan (95.8%) and Bishah (91.9%).

### 3.8. Forecasted National Trend

**Figure 5:**
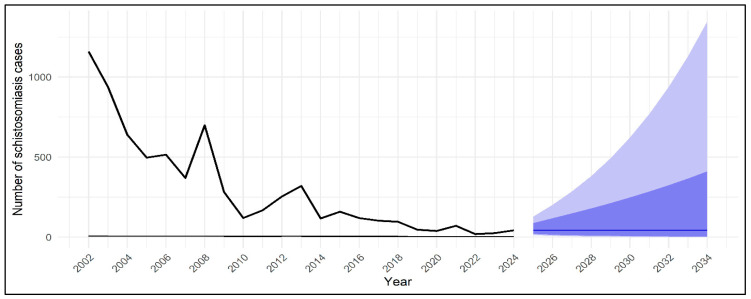
Forecasted national schistosomiasis cases in Saudi Arabia using ARIMA modeling. Observed annual schistosomiasis case counts from 2002 to 2024 are shown by the solid black line. Forecasts were generated using an autoregressive integrated moving average (ARIMA) model fitted to log-transformed case counts, with zero used as the baseline reference to reflect the theoretical lower bound of case detection in an elimination-phase setting. The projected mean trend is displayed by the central forecast line, while the shaded areas represent prediction intervals: the darker blue band indicates the 80% prediction interval and the lighter blue outer band indicates the 95% prediction interval, reflecting increasing uncertainty with longer forecast horizons.

National ARIMA-based forecasts are presented in Figure 5. Projections indicated continued low-level detection of schistosomiasis cases over the forecast horizon. The projected mean remained stable, and both the 80% and 95% prediction intervals are shown. The forecasts do not indicate a return to the higher incidence levels that were observed during the early years of the study.

### 3.9. Regional Forecasts

**Figure 6:**
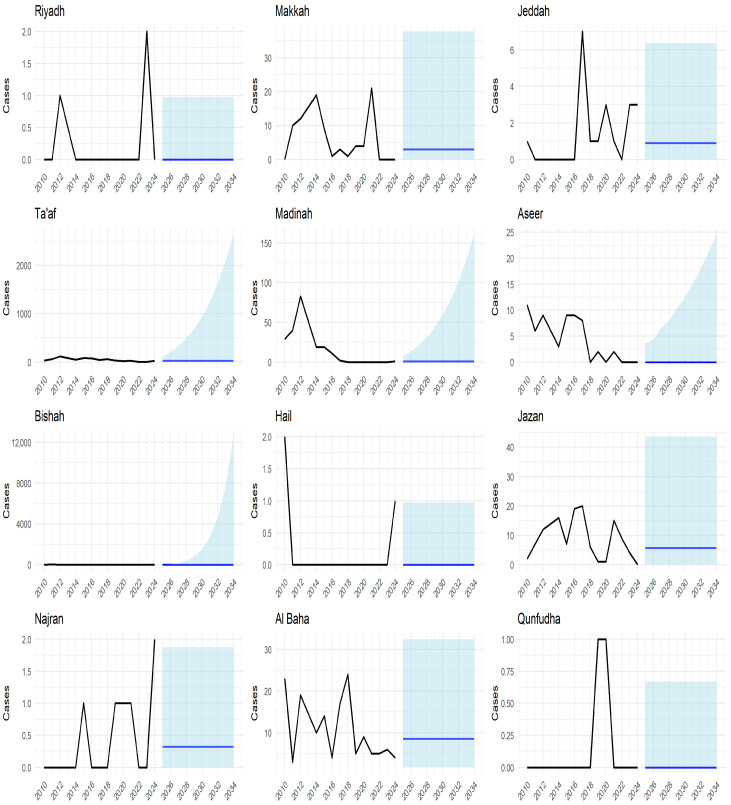
Regional ARIMA-based projections of schistosomiasis cases in selected health regions of Saudi Arabia since 2010. Each panel represents a selected health region. The black line shows observed annual schistosomiasis case counts during the historical period (2010–2024). The transition from the observed data to the shaded area indicates the start of the forecast period. The blue line represents the projected mean number of cases generated by the ARIMA model, while the shaded band indicates the 95% prediction interval, reflecting uncertainty around the forecast. The y-axis scale varies across panels to accommodate differences in case magnitude between regions.

Regional ARIMA projections are shown in Figure 6 and demonstrate heterogeneous but stable trajectories across selected health regions. Across regions, forecasted mean case counts remained low throughout the projection period, with most trajectories fluctuating close to zero. Regions with higher historical case counts showed slightly higher projected means and wider prediction intervals, whereas regions with fewer reported cases exhibited flatter projections with narrow uncertainty bands.

Importantly, no region demonstrated a sustained upward trajectory or widening trend suggestive of re-emergence during the forecast period. Overall, the regional projections are consistent with continued low-level detection and stable patterns across health regions during the elimination-maintenance phase.

## 4. Discussion

This study provides a comprehensive long-term assessment of schistosomiasis epidemiology in Saudi Arabia using national surveillance data covering more than two decades. The findings demonstrate a sustained decline in reported cases and incidence, with joinpoint analysis identifying a significant change in trend around 2010, after which case numbers declined more steeply and stabilized at low levels. Collectively, these patterns indicate a transition from historical endemic transmission to an elimination-maintenance phase characterized by sporadic and focal case detection rather than widespread transmission.

This epidemiological transition aligns with the strategic direction of the World Health Organization (WHO) Neglected Tropical Diseases Road Map 2021–2030, which emphasizes progression from morbidity control toward interruption of transmission, impact-based monitoring, and integration of NTD programs into national health systems [3]. In elimination-phase settings, low case numbers, temporal fluctuations, and geographic heterogeneity are expected, and careful interpretation of surveillance data is required to distinguish sustained progress from re-emergence [3,4]. The stable post-2010 trends observed in Saudi Arabia are consistent with these expectations.

The observed decline reflects the cumulative impact of long-standing national control efforts, including environmental modification, snail control, expanded diagnostic capacity, and sustained access to praziquantel. The identification of a single major change point suggests a gradual, programmatically driven transition rather than an abrupt interruption of transmission. The absence of subsequent change points or sustained upward trends supports the durability of these gains and argues against ongoing widespread transmission, consistent with WHO criteria for countries approaching elimination [3].

In recent years, incidence remained consistently below one case per 100,000 population, accompanied by increasing spatial focality. Such patterns are typical of elimination-maintenance settings, where remaining cases often reflect residual transmission in limited ecological niches, occupational or travel-related exposure, or imported infections rather than continuous community-level transmission [2,9]. However, the national schistosomiasis surveillance data used in this study does not include individual-level exposure or travel histories, limiting the ability to distinguish imported from locally acquired infections. Unlike malaria surveillance in Saudi Arabia, which routinely captures importation status, schistosomiasis reporting remains aggregated, emphasizing the need for cautious interpretation and targeted surveillance [3].

Analysis of clinical subtypes further supports this transition. Intestinal schistosomiasis accounted for the majority of reported cases throughout the study period and an even higher proportion in recent years. This predominance reflects historical transmission patterns in western and southwestern Saudi Arabia, where *S. mansoni* was dominant, and aligns with earlier studies documenting declines in urogenital schistosomiasis following sustained control efforts [7,13]. The rarity of mixed infections and declining contribution of urinary schistosomiasis are consistent with reduced transmission intensity and fragmented transmission cycles typical of late-phase elimination settings [9].

A key finding is the near absence of schistosomiasis among pediatric populations after 2010. Children younger than 15 years accounted for only a very small proportion of reported cases, contrasting sharply with earlier periods when pediatric infections were common during active transmission. Hospital-based studies previously documented pediatric schistosomiasis in Saudi Arabia during periods of higher endemicity [14]. The marked reduction in pediatric cases observed in this study strongly suggests interruption of local transmission, a pattern widely recognized as a hallmark of elimination-phase epidemiology [25].

On the contrary, cases became increasingly concentrated among adults, particularly those aged 15–39 years, and among non-Saudi nationals. The shift in nationality patterns observed after 2009 likely reflects sustained reductions in local transmission among Saudi nationals following long-term control efforts, alongside an increasing contribution of infections among non-Saudi adults, including expatriates originating from endemic countries, and enhanced surveillance sensitivity during the elimination-maintenance phase. This demographic shift suggests alternative exposure pathways, including occupational exposure and population mobility, rather than ongoing community-level transmission. Similar patterns have been documented internationally as countries approach elimination, where remaining infections are increasingly detected among mobile adult populations rather than locally exposed children [2,3]. These findings support WHO recommendations for people-centered, risk-based surveillance approaches in elimination settings [3].

Despite low national incidence, schistosomiasis in Saudi Arabia remains geographically heterogeneous. Since 2010, most cases were concentrated in a limited number of western and southwestern regions, particularly Ta’if, Al Baha, Jazan, and Madinah, corresponding to areas of historical endemicity and ecological suitability for intermediate host snails. Localized studies from Al Baha documented measurable prevalence as recently as the early 2010s, highlighting the persistence of focal transmission in ecologically favorable settings despite national declines [13]. In contrast, many regions reported few or no cases over extended periods, indicating sustained interruption of transmission. This pattern highlights the need for region-specific surveillance in addition to the uniform national interventions. These findings highlight the continuing influence of environmental and ecological factors, such as freshwater availability, irrigation practices, and snail habitats, in shaping residual transmission risk. Future surveillance strategies would benefit from integrating environmental and ecological data to better characterize sub-regional heterogeneity beyond administrative boundaries.

Saudi Arabia’s trajectory is consistent with international and regional elimination experiences. Oman provides a relevant regional benchmark, having documented sustained declines in *S. mansoni* transmission and no reported intestinal schistosomiasis cases in recent years [10,11]. While direct comparison is limited by differences in geography and surveillance systems, Oman’s experience demonstrates the feasibility of elimination in the Gulf region. Saudi Arabia’s progress is particularly notable given its larger land area, population size, and ecological diversity.

Recent national analyses further support these findings. Ahmed and Alharbi [15], examining schistosomiasis trends from 2021 to 2023, reported continued low-level detection and emphasized the importance of sustained surveillance during the elimination phase. By extending the temporal scope to over two decades and applying formal change-point detection and forecasting methods, the present study strengthens evidence that observed declines represent a durable epidemiological transition rather than short-term fluctuation.

Time-series forecasting indicates continued low-level detection without evidence of resurgence at either the national or regional level. Although forecasting in elimination settings must be interpreted cautiously, the absence of upward trends aligns with WHO expectations for countries operating within an elimination-maintenance phase [3,4].

Several limitations should be acknowledged. Surveillance data were aggregated and derived from routine reporting systems, which may be subject to underreporting or variation in diagnostic practices. Individual-level exposure and travel histories were unavailable, limiting differentiation between imported and residual local infections. Regional reporting was also incomplete in earlier years. Nevertheless, the consistency of findings across multiple indicators and analytical approaches supports the robustness of the conclusions.

## 5. Conclusions

Schistosomiasis in Saudi Arabia has undergone a clear epidemiological transition, characterized by sustained reductions in incidence, near absence of pediatric cases, and geographically focal persistence. Integration of long-term national surveillance data with change-point analysis and forecasting provides strong evidence that the Kingdom is operating within an elimination-maintenance phase, consistent with the goals of the WHO NTD Road Map 2021–2030. Continued targeted surveillance, particularly in historically endemic regions and among adult and mobile populations, will be essential to consolidate gains, support impact-based monitoring, and prevent re-establishment of transmission.

## Figures and Tables

**Table 1:** National schistosomiasis cases, incidence, clinical subtype, and demographic characteristics in Saudi Arabia (2002–2024).

Year	Total Cases	Incidence per 100,000 *	Clinical Schistosomiasis Subtypes			Sex		Nationality		Age Groups			
			Urinary	Intestinal	Mixed	Male	Female	Saudi	Non-Saudi	<5	5–14	15–39	≥40
**2002**	1159	5.5	687	459	13	890	269	670	489	-	-	-	-
**2003**	938	4.5	434	501	3	773	165	543	395	-	-	-	-
**2004**	639	2.9	277	360	2	524	115	391	245	-	-	-	-
**2005**	497	2.2	203	293	1	408	89	352	145	-	-	-	-
**2006**	515	2.2	172	343	0	424	91	324	191	1	162	283	69
**2007**	370	1.52	125	245	0	311	59	267	103	1	136	184	49
**2008**	699	2.78	172	524	3	574	125	388	311	2	155	434	108
**2009**	282	1.1	44	234	4	258	24	129	153	0	28	204	50
**2010**	120	0.50	14	104	2	102	18	46	74	1	15	82	22
**2011**	168	0.60	27	141	0	148	20	31	137	0	11	130	27
**2012**	254	0.90	35	219	0	150	104	48	206	0	15	214	25
**2013**	320	1.07	18	302	0	-	-	-	-	-	-	-	-
**2014**	117	0.38	27	90	0	92	25	37	80	0	21	84	12
**2015**	159	0.50	19	140	0	139	20	59	100	1	30	113	15
**2016**	119	0.37	32	87	0	105	14	27	92	0	15	93	11
**2017**	103	0.32	35	68	0	99	4	23	80	0	7	87	9
**2018**	96	0.29	9	87	0	89	7	26	70	0	21	68	7
**2019**	47	0.14	9	38	0	40	7	12	35	0	5	37	5
**2020**	39	0.12	8	31	0	33	6	13	26	0	6	31	2
**2021**	71	0.23	20	51	0	58	13	19	52	2	10	42	17
**2022**	19	0.06	10	7	2	14	5	11	8	1	5	11	2
**2023**	25	0.07	10	13	2	21	4	10	15	2	4	18	1
**2024**	43	0.12	12	26	5	39	4	8	35	0	2	35	6

* Incidence values are reported as published by the Saudi Ministry of Health. - Indicates data not available.

**Table 2:** Regional distribution, clinical subtype, and demographic characteristics of schistosomiasis cases in Saudi Arabia, 2010–2024.

Region	Total Cases	Clinical Schistosomiasis Subtypes			Sex		Nationality		Age Groups			
Region	Total Cases	Urinary	Intestinal	Mixed	Male	Female	Saudi	Non-Saudi	<5	5–14	15–39	≥40
**Ta’if**	639	215	421	3	548	91	203	436	1	10	472	156
**Madinah**	204	8	196	0	181	23	184	20	0	0	198	6
**Al Baha**	148	42	106	0	120	28	111	37	1	39	92	16
**Jazan**	119	11	108	0	104	15	114	5	2	34	77	6
**Makkah**	84	24	58	2	70	14	24	60	0	5	32	47
**Bishah**	74	30	44	0	61	13	68	6	0	12	52	10
**Aseer**	59	21	38	0	52	7	49	10	0	9	41	9
**Jeddah**	20	3	17	0	18	2	6	14	0	0	20	0
Eastern Province *	19	4	15	0	17	2	3	16	1	1	15	2
**Najran**	6	2	4	0	4	2	3	3	0	0	3	3
**Riyadh**	3	1	2	0	3	0	2	1	0	0	3	0
**Hail**	3	1	2	0	2	1	2	1	0	1	2	0
**Qunfudha**	2	1	1	0	2	0	2	0	0	0	2	0

Notes: * Eastern Province: regional surveillance data were available from 2018 onward; earlier years were not reported and therefore not included.

## Data Availability

The data supporting the findings of this study are available from publicly accessible reports of the Ministry of Health of Saudi Arabia. The datasets analyzed during the current study are available at: https://www.moh.gov.sa/en/Ministry/Statistics/book/Pages/default.aspx, accessed on 10 November 2025.

## Abbreviations

The following abbreviations are used in this manuscript:

WHO	World Health Organization
NTDs	Neglected Tropical Diseases
MoH	Ministry of Health
ARIMA	Autoregressive Integrated Moving Average
